# Preoperative sleep quality affects postoperative pain and function after total joint arthroplasty: a prospective cohort study

**DOI:** 10.1186/s13018-019-1446-9

**Published:** 2019-11-21

**Authors:** Ze-Yu Luo, Ling-Li Li, Duan Wang, Hao-Yang Wang, Fu-Xing Pei, Zong-Ke Zhou

**Affiliations:** Department of Orthopedics, West China Hospital/West China School of Medicine, Sichuan University, 37# Wuhou Guoxue Road, Chengdu, 610041 People’s Republic of China

**Keywords:** Total joint arthroplasty, Pain, Sleep, Clinical function

## Abstract

**Background:**

The relationship between preoperative sleep quality and postoperative clinical outcomes after total joint arthroplasty (TJA) is unclear. We performed a prospective cohort study to determine whether preoperative sleep quality was correlated with postoperative outcomes after TJA.

**Methods:**

In this prospective cohort study, 994 patients underwent TJA. Preoperative sleep measures included scores on the Pittsburgh Sleep Quality Index (PSQI), the Epworth Sleepiness Scale (ESS), and a ten-point sleep quality scale. The primary study outcome measured was the visual analog scale (VAS) pain score to 12 weeks postoperation. The consumption of analgesic rescue drugs (oxycodone and parecoxib) and postoperative length of stay (LOS) were recorded. We also measured functional parameters, including range of motion (ROM), Knee Society Score (KSS), and Harris hip score (HHS).

**Results:**

The mean age for total knee and hip arthroplasties was 64.28 and 54.85 years, respectively. The PSQI scores were significantly correlated with nocturnal and active pain scores and ROM and functional scores from postoperative day 1 (POD1) to POD3. In addition, significant correlation was noted between the correlation between the active pain scores and ESS scores in the TKA group at postoperative 3 months. The consumption of analgesics after joint arthroplasty was significantly correlated with the PSQI scores. Moreover, significant correlations were noted between the sleep parameters and postoperative length of hospital stay (LOS).

**Conclusion:**

Preoperative sleep parameters were correlated with clinical outcomes (i.e., pain, ROM, function, and LOS) after TJA. Clinicians should assess the sleep quality and improve it before TJA.

## Introduction

More than 21 million adults in the US have osteoarthritis (OA) [1]. These patients experience a worsening in their quality of life and a decreased ability to perform activities of daily living [2]. Studies have reported that patients with OA have decreased sleep quality in association with increased night pain, which correlates with radiographic severity of disease [3, 4]. Pain has been identified as a cause of poor sleep quality. Poor sleep quality has been associated with increased pain perception [5].

Total joint arthroplasty (TJA) has proven to be a surgical option for patients with advanced osteoarthritis in terms of improved function and pain relief [6]. Both primary total hip (THA) and knee (TKA) arthroplasties can relieve pain, restore function, and increase mobility to levels observed in the general population [7]. However, a previous study has shown that patients’ sleep quality is impaired for 3 months after joint replacement and further affects patients’ rapid recovery after surgery [8].

Several studies have investigated the influence of different interventions on postoperative sleep quality [9–11]. The relationship between postoperative sleep quality and postoperative rehabilitation and did not further explore the influence of preoperative sleep quality in patients. Although many previous studies have investigated the influence of postoperative sleep quantity on rehabilitation after total joint replacement, the correlations between the preoperative sleep quality and postoperative clinical outcomes (i.e., pain and function) are unclear. Studies of healthy, pain-free volunteers have consistently shown that sleep continuity disturbance impairs endogenous pain-inhibitory function and increases spontaneous pain, supporting a possible pathophysiologic role of sleep disturbance in pain [12–14]. One study found that a presurgery extended time-in-bed and the associated increase in sleep time in short-sleeping patients scheduled for undergoing joint replacement resulted in reduced postsurgery pain ratings and opioid medication use [15]. However, they included only seven patients in the treatment group, which may not be powered to detect a significant difference.

We, therefore, performed a prospective cohort study to determine whether presurgery sleep quality was correlated with postoperative pain, function, complications, and length of hospital stay (LOS) after TKA and THA.

## Methods

Approval was obtained from the Clinical Trials and Biomedical Ethics Committee of West China Hospital, and written informed consent was obtained from all participants. This study was registered in the Chinese Clinical Trial Registry (ChiCTR1900022357).

### Patients

From March 2017 to March 2018, patients who were scheduled to undergo primary unilateral TJA were eligible for study enrollment. Patients were excluded based on the following criteria: primary sleep disorders (insomnia, apnea, and restless legs), mental disorders (depression, anxiety), chronic uncontrolled medical illnesses beyond their joint disease (preoperative hepatic or renal dysfunction and serious cardiac and/or cerebrovascular comorbidities), planned revision surgery, bilateral arthroplasty, and refusal to participate.

### Surgical procedure and pain management

All total hip and knee replacement surgeries were performed under general anesthesia by two orthopedic surgeons. The patients were sent to the inpatient orthopedic unit where they spent 3–4 days before being discharged to continue their recovery at home after the surgery. The lights in the inpatient unit were dimmed between 10 pm and 6 am, and patient doors were closed. Six beds were placed in each inpatient unit. No attempt was made to manage the sleep quality for this group of patients.

Pain management [16] was used for pain control in all patients according to the standard practice at our institution, as described by Lei et al. [17]. Multimodal oral analgesic drugs (diclofenac 50 mg q 12 h, Novartis Biomedical Research Co., Ltd., Switzerland, and pregabalin 75 mg q 8 h, Pfizer Pharmaceuticals Co., Ltd., USA) were administered for preemptive analgesia 1 day before surgery. This analgesic regimen was repeated when patients resumed postoperative oral intake. When patients reported pain greater than 4 on a 0–10 visual analog scale (VAS), oral oxycodone (10 mg q 8 h) was utilized. An intramuscular injection of parecoxib (40 mg) was used if a patient claimed severe pain greater than 6 on the VAS [17].

Prophylaxis against venous thromboembolism was administered following the standard practice at our institution [18, 19]. An examination of the clinical symptoms of deep vein thrombosis (DVT) in all patients was conducted according to the standard practice at our institution [18]. During hospitalization, the patients were examined daily for any clinical symptoms of DVT. Ultrasound examinations were also performed routinely at specific time points (preoperatively, at discharge, and at the 2-week and 3-month follow-ups). If a patient was symptomatic, an ultrasound examination was performed immediately. A contrast-enhanced chest spiral computed tomography was performed immediately for any clinical signs of pulmonary embolism (PE) [18, 20]. In addition, other complications including superficial infection, hematoma, and wound secretion were also diagnosed according to the standardized list and definitions [21, 22].

### Sleep quality assessments

Sleep quality was evaluated by 2 separate assessments (ZY. L. and D. W.). Sleep quality can be measured by using three subjective methods. The patients were required to complete the Pittsburgh Sleep Quality Index (PSQI), a self-rated questionnaire that assesses sleep quality and disturbances over a 1-month time interval prior to admission [9, 23]. Nineteen individual items generate 7 “component” scores: subjective sleep quality, sleep latency, sleep duration, habitual sleep efficiency, sleep disturbances, use of sleeping medication, and daytime dysfunction. The sum of scores for these 7 components yields 1 global score, with a maximum score of 21. A higher score indicates worse sleep dysfunction, and a score greater than 5 indicates poor sleep quality [8]. Daytime sleepiness assessment was assessed using the Epworth Sleepiness Scale (ESS) [24]. The ESS is a standardized, validated questionnaire for assessing daytime sleepiness. It contains 8 questions that ask respondents to assess on a 4-point scale the typical opportunities for them to fall asleep or doze off during various daily activities. The Epworth sleep scale score ranged from 0 to 24 (sum of 8 scores, 0–3), with higher scores indicating a greater propensity for daytime sleepiness. In general, ESS scores can be interpreted as follows: 0–5 lower normal daytime sleepiness, 6–10 higher normal daytime sleepiness, 11–12 mild excessive daytime sleepiness, 13–15 moderate excessive daytime sleepiness, and 16–24 severe excessive daytime sleepiness [25]. Finally, we used a ten-point scale to measure the patient’s sleep quality, defined on a scale from 0 (extremely poor sleep quality) to 10 (extremely good sleep quality). The higher the score was, the better the quality of sleep.

### Clinical outcomes

The primary study outcome measured was the VAS pain score, defined on a scale from 0 (no pain) to 10 (severe pain). In particular, we recorded active and nocturnal VAS pain scores, respectively, which can provide additional useful and detailed information about patients’ pain sensations before and after surgery. Night pain was assessed in the morning by nursing staff. In addition, the consumption of analgesic rescue drugs (oxycodone and parecoxib) was recorded, and the doses of oxycodone were expressed in morphine milligram equivalents. We also measured functional parameters, including ROM, Knee Society Score (KSS), and Harris hip score (HHS). In addition, all patients were required to complete a 10-point satisfaction questionnaire upon discharge to assess their satisfaction level, defined on a scale from 0 (very dissatisfied) to 10 (extremely satisfied) [26, 27], and the postoperative LOS was recorded. At our institution, patients undergoing arthroplasty are prospectively followed up at 2 weeks and 12 weeks to record their clinical outcomes. For enrolled patients who did not return to their postoperative outpatient appointments, the results were obtained by telephone at the appropriate follow-up points. All complications were also recorded during the first 3 postoperative months.

### Statistical analysis

Distributions of demographic data, baseline data, and primary and secondary outcomes were assessed using measures of central tendency (mean, standard deviation) for quantitative variables and with percentages for qualitative variables. Pearson correlations were performed between the PSQI, ESS, and sleep quality scores with the VAS scores, dose of analgesic rescue drugs, ROM, HHS, KSS, satisfaction score, and LOS. Spearman correlations were also performed between sleep parameters and the number of patients requiring analgesic rescue drugs. An independent sample *t* test was used to compare patients with and without complications against their PSQI, ESS, and sleep quality measures. All data analyses were performed using SPSS for Windows, Version 19.0 (SPSS Inc., Chicago, IL, USA). Significance was set at *P* < 0.05.

## Results

### Patient demographics and perioperative characteristics

From March 2017 to March 2018, 691 and 724 patients were scheduled to undergo total knee and hip replacement, respectively, at our institution. Among these patients, 191 and 187 were ineligible, while 29 and 14 refuse to participate. Hence, 471 and 523 patients were enrolled in the cohort. No patients were lost or excluded during the follow-up.

The mean patient age (and standard deviation) for those that underwent TKA and THA was 64.28 ± 9.68 and 54.85 ± 13.20 years, respectively. A total of 114 (24.2%) and 289 (55.2%) patients were men among the TKA and THA patients, respectively. The mean patient body mass index (BMI) was 25.81 ± 3.57 and 24.10 ± 3.97 kg/m^2^, respectively (Table 1). Preoperative sleep parameters (i.e., PSQI, ESS, sleep quality scores) were 10.27 ± 3.77, 7.70 ± 2.31, and 7.86 ± 1.39 for TKA patients, and 10.11 ± 3.56, 7.36 ± 2.41, and 8.19 ± 3.72 for the THA patients (Table 1).

**Table 1:** Baseline, perioperative, and demographic characteristics of the patients

Variable	TKA (*N* = 471)	THA (*N* = 523)
Demographic characteristics		
Age (years)	64.28 ± 9.68	54.85 ± 13.20
Male [no.[%] of patients]	114 (24.2%)	289 (55.2%)
Height (m)	1.57 ± 0.07	1.61 ± 0.08
Weight (kg)	63.90 ± 10.19	62.71 ± 12.01
BMI (kg/m^2^)	25.81 ± 3.57	24.10 ± 3.97
Operated side [no.[%] of patients]		
Right	221 (46.9%)	244 (46.6%)
Left	250 (53.1%)	279 (53.4%)
Comorbidities [no.[%] of patients]		
Obstructive sleep apnea	109 (23.1%)	123 (23.5%)
COPD	56 (11.8%)	65 (12.4%)
Hypertension	127 (26.9%)	132 (25.2%)
Diabetes mellitus	75 (15.9%)	87 (16.6%)
Preop. pain and function		
ROM, °	92.15 ± 13.60	89.29 ± 15.71
VAS-night	3.84 ± 1.41	3.20 ± 1.50
VAS-ambulation	6.37 ± 1.73	5.33 ± 1.02
HHS (points)	–	53.07 ± 13.33
KSS (points)	52.98 ± 13.14	
Sleep parameters		
PSQI	10.27 ± 3.77	10.11 ± 3.56
ESS	7.70 ± 2.31	7.36 ± 2.41
Sleep quality	7.86 ± 1.39	8.19 ± 3.72

*TKA* total knee arthroplasty, *THA* total hip arthroplasty, *BMI* body mass index, *COPD* chronic obstruct pulmonary disease, *ROM* range of motion, *VAS* visual analog scale, *HHS* Harris hip score, *KSS* knee society score, *PSQI* the Pittsburgh Sleep Quality Index, *ESS* Epworth Sleepiness Scale

### Pain and clinical outcomes

Postoperative pain and clinical outcomes are reported in Table 2. Notably, the nocturnal and active pain scores in the TKA group were 3.14 ± 1.41 and 4.99 ± 1.73 on postoperative day (POD) 1, and the pain was gradually relieved 3 days, 14 days, and 3 months after surgery. In addition, the pain scores in the THA group showed a similar trend than the TKA group. The average ROM was 98.11 ± 11.29 and 97.69 ± 7.47 for the TKA and THA groups on POD1 and 110.20 ± 5.96 and 110.13 ± 6.07 on POD3, respectively (Table 2). The number of patients requiring parecoxib and oxycodone (morphine milligram equivalents) is shown in Table 2. No PE or DVT was observed in any group (Table 3). The average satisfaction level of the two groups after joint replacement reached 8.9 points. The average postoperative LOS in the knee replacement group was 4.21 ± 2.23 days, while that in the hip replacement group was 4.05 ± 2.51 days (Table 3).

**Table 2:** Pain and clinical function outcome postoperation

Variable	TKA (*N* = 471)	THA (*N* = 523)
POD1		
VAS-night	3.14 ± 1.41	2.79 ± 1.71
VAS-ambulation	4.99 ± 1.73	4.11 ± 1.88
ROM°	98.11 ± 11.29	97.69 ± 7.47
HHS (points)	–	58.38 ± 13.51
KSS (points)	54.03 ± 13.09	–
POD3		
VAS-night	2.67 ± 1.29	2.26 ± 1.35
VAS-ambulation	3.93 ± 1.54	2.67 ± 1.66
ROM°	110.20 ± 5.96	110.13 ± 6.07
HHS (points)	–	67.00 ± 11.25
KSS (points)	61.49 ± 11.52	–
POD14		
VAS-night	1.52 ± 0.93	1.60 ± 1.06
VAS-ambulation	2.18 ± 1.11	2.23 ± 1.22
ROM°	112.34 ± 4.63	113.08 ± 4.83
HHS (points)	–	76.65 ± 8.56
KSS (points)	77.32 ± 7.46	–
POM3		
VAS-night	0.98 ± 0.81	1.04 ± 0.83
VAS-ambulation	1.48 ± 1.13	1.54 ± 1.10
ROM°	110.85 ± 6.65	112.14 ± 6.73
HHS (points)	–	83.23 ± 4.64
KSS (points)	81.65 ± 4.51	–
Oxycodone		
Number of patients requiring	68	31
Dose (morphine eq.)	7.71 ± 15.39	3.71 ± 9.02
Parecoxib		
Number of patients requiring	52	27
Dose	5.90 ± 14.64	3.54 ± 11.63

*TKA* total knee arthroplasty, *THA* total hip arthroplasty, *ROM* range of motion, *VAS* visual analog scale, *HHS* Harris hip score, *KSS* knee society score, *POD* postoperative day, *POM* postoperative month

**Table 3:** Outcomes regarding adverse events, length of hospital stay, and satisfaction level

Variable	TKA (*N* = 471)	THA (*N* = 523)
Symptomatic DVT	0	0
Asymptomatic DVT	11	14
CMVT	32	33
PE	0	0
Superficial infection	3	2
Haematoma	5	6
Wound secretion	4	6
Satisfaction	8.92 ± 0.80	8.98 ± 0.81
LOS	4.21 ± 2.23	4.05 ± 2.51

*TKA* total knee arthroplasty, *THA* total hip arthroplasty, *DVT* deep vein thrombosis, *CMVT* calf muscular vein thrombosis, *PE* pulmonary embolism, *LOS* length of stay

### Correlation between sleep and clinical parameters

The correlations between the sleep parameters and postoperative pain, ROM, postoperative LOS, and clinical function outcomes after TKA are presented in Table 4. Before TKA, the PSQI scores were significantly correlated with increased nocturnal (*r* = 0.123, *P* = 0.008) and active (*r* = 0.118, *P* = 0.010) pain scores. In addition, the PSQI scores were significantly correlated with nocturnal (*r* = 0.098, *P* = 0.034) and active (*r* = 0.127, *P* = 0.006) pain scores and ROM (*r* = − 0.149, *P* = 0.001) and KSS (*r* = − 0.209, *P* = 0.001) on POD1. Similarly, sleep quality was significantly correlated with active pain scores (*r* = − 0.099, *P* = 0.031) and ROM (*r* = 0.129, *P* = 0.005) on POD1. There was also a significant correlation between the PSQI and active pain scores measures on POD3 (*r* = 0.119, *P* = 0.009). However, no significant correlations were noted between the sleep parameters and functional outcomes on POD14. In addition, a negative correlation between the active pain scores and ESS scores was noted on POM3 (*r* = − 0.091, *P* = 0.049).

**Table 4:** Sleep parameters compared with patient reported pain, ROM, and clinical function after surgery

Variable	TKA						THA					
	PSQI		ESS		Sleep quality		PSQI		ESS		Sleep quality	
	r value	*p* value	*r* value	*p* value	*r* value	*p* value	*r* value	*p* value	*r* value	*p* value	*r* value	*p* value
Preoperation												
VAS-night	0.123	*0.008*	− 0.056	0.226	− 0.087	0.060	0.111	*0.011*	0.071	0.107	0.049	0.264
VAS-ambulation	0.118	*0.010*	0.039	0.403	0.005	0.916	0.120	*0.006*	0.033	0.451	− 0.006	0.898
ROM°	− 0.033	0.470	0.027	0.553	0.051	0.269	− 0.038	0.381	− 0.034	0.432	− 0.011	0.798
KSS/HHS	− 0.030	0.510	0.020	0.665	− 0.007	0.887	− 0.134	*0.002*	− 0.098	*0.025*	0.096	*0.028*
POD1												
VAS-night	0.098	*0.034*	0.060	0.195	− 0.037	0.425	0.120	*0.006*	0.059	0.176	0.020	0.655
VAS-ambulation	0.127	*0.006*	0.018	0.692	− 0.099	*0.031*	0.088	*0.043*	0.060	0.169	− 0.006	0.893
ROM°	− 0.149	*0.001*	0.040	0.382	0.129	*0.005*	− 0.065	0.140	− 0.093	*0.034*	− 0.037	0.402
KSS/HHS	− 0.209	*0.001*	− 0.101	*0.029*	0.076	0.100	− 0.186	*0.000*	− 0.081	0.065	0.086	0.050
POD3												
VAS-night	0.111	*0.016*	0.072	0.118	− 0.077	0.094	0.133	*0.002*	0.019	0.662	− 0.095	*0.030*
VAS-ambulation	0.119	*0.009*	0.108	*0.019*	− 0.049	0.289	0.101	*0.021*	− 0.014	0.745	− 0.008	0.849
ROM°	0.031	0.498	0.027	0.558	0.029	0.527	− 0.002	0.969	0.000	0.994	− 0.058	0.185
KSS/HHS	− 0.054	0.239	− 0.088	0.057	− 0.034	0.461	− 0.123	*0.005*	− 0.029	0.507	0.071	0.103
POD14												
VAS-night	− 0.057	0.220	0.004	0.934	0.072	0.117	0.106	*0.016*	0.096	*0.028*	0.017	0.702
VAS-ambulation	0.029	0.536	0.009	0.840	− 0.043	0.352	0.033	0.445	0.006	0.895	0.037	0.399
ROM°	0.005	0.918	− 0.005	0.918	− 0.004	0.934	− 0.032	0.461	− 0.119	*0.007*	− 0.021	0.627
KSS/HHS	0.042	0.366	0.003	0.954	− 0.022	0.636	− 0.059	0.176	− 0.028	0.527	− 0.053	0.224
POM3												
VAS-night	0.048	0.295	− 0.027	0.552	− 0.054	0.238	− 0.051	0.244	− 0.004	0.923	− 0.050	0.255
VAS-ambulation	− 0.072	0.120	− 0.091	*0.049*	0.044	0.345	− 0.048	0.270	− 0.030	0.491	− 0.046	0.295
ROM°	− 0.067	0.145	− 0.014	0.754	− 0.004	0.934	− 0.028	0.518	− 0.025	0.573	0.079	0.071
KSS/HHS	− 0.014	0.757	− 0.035	0.443	0.039	0.402	0.017	0.704	0.059	0.178	0.012	0.778
Satisfaction	0.011	0.812	0.009	0.840	0.022	0.636	− 0.011	0.810	0.073	0.093	0.010	0.828
LOS	0.367	*0.000*	0.097	*0.036*	− 0.171	*0.000*	0.268	*0.000*	0.115	*0.008*	− 0.095	*0.030*

*TKA* total knee arthroplasty, *THA* total hip arthroplasty, *ROM* range of motion, *VAS* visual analog scale, *HHS* Harris hip score, *KSS* knee society score, *POD* postoperative day, *POM* postoperative month, *PSQI* the Pittsburgh Sleep Quality Index, *ESS* Epworth Sleepiness Scale, *LOS* length of stay. Significant *p* values appear in italics

The correlations between the sleep parameters and postoperative pain, ROM, postoperative LOS, and clinical function outcomes after THA are presented in Table 4. The PSQI scores were significantly correlated with increased nocturnal (*r* = 0.111, *P* = 0.011) and active (*r* = 0.120, *P* = 0.006) pain scores and the HHS (*r* = − 0.134, *P* = 0.002) before THA. Similarly, the ESS (*r* = − 0.134, *P* = 0.002) and sleep quantity (*r* = − 0.098, *P* = 0.025) measures were also noted to be significantly correlated with the HHS before THA. In addition, the PSQI scores significantly correlated with the pain scores on POD1 and POD3.

The correlations between sleep parameters and analgesic use are documented in Table 5. The average doses of parecoxib after knee arthroplasty were significantly correlated with the PSQI scores (*r* = 0.114, *P* = 0.013). In addition, the number of patients requiring parecoxib was also correlated with the PSQI scores in the THA group (*r* = 0.086, *P* = 0.048). However, no significant correlations were noted between the other sleep parameters and consumption of the analgesics.

**Table 5:** Sleep parameters compared with the requirement of the rescue treatment after surgery

Variable	TKA						THA					
	PSQI		ESS		Sleep quality		PSQI		ESS		Sleep quality	
	*r* value	*p* value	*r* value	*p* value	*r* value	*p* value	*r* value	*p* value	*r* value	*p* value	*r* value	*p* value
Oxycodone												
Number of patients requiring	− 0.059	0.205	− 0.044	0.338	− 0.034	0.467	0.052	0.239	0.037	0.403	− 0.035	0.423
Dose	− 0.026	0.568	− 0.030	0.517	− 0.041	0.376	0.058	0.183	0.039	0.377	− 0.027	0.540
Parecoxib												
Number of patients requiring	0.090	0.052	− 0.007	0.887	− 0.039	0.396	0.086	*0.048*	0.077	0.078	− 0.073	0.095
Dose	0.114	*0.013*	− 0.014	0.768	− 0.057	0.216	0.074	0.091	0.079	0.071	− 0.030	0.489

*TKA* total knee arthroplasty, *THA* total hip arthroplasty, *PSQI* the Pittsburgh Sleep Quality Index, *ESS* Epworth Sleepiness Scale. Significant *p* values appear in italics

Moreover, significant correlations were noted between the sleep parameters and postoperative LOS, which indicated that increased postoperative LOS correlated with poor sleep quality (Table 4). However, there were no significant correlations between sleep parameters and perioperative complications, and postoperative satisfaction levels were also not significantly correlated with sleep parameters (Table 6).

**Table 6:** Sleep parameters compared with complications after TJA

	TKA									THA								
	PSQI			ESS			Sleep quality			PSQI			ESS			Sleep quality		
	Complication	None	p value	Complication	None	p value	Complication	None	p value	Complication	None	p value	Complication	None	p value	Complication	None	p value
Asymptomatic DVT	8.90 ± 3.53	10.29 ± 3.77	0.228	7.72 ± 2.10	7.70 ± 2.31	0.972	7.45 ± 0.82	7.87 ± 1.40	0.326	11.14 ± 4.03	10.09 ± 3.55	0.276	7.92 ± 3.02	7.35 ± 2.39	0.380	8.14 ± 1.46	8.19 ± 3.77	0.959
CMVT	11.38 ± 3.31	10.17 ± 3.79	0.073	8.05 ± 2.46	7.67 ± 2.30	0.352	8.00 ± 1.45	7.85 ± 1.38	0.549	9.18 ± 3.91	10.18 ± 3.53	0.119	7.36 ± 2.47	7.36 ± 2.41	0.989	8.15 ± 1.25	8.19 ± 3.84	0.947
Superficial infection	10.66 ± 2.08	10.26 ± 3.78	0.854	8.00 ± 4.00	7.70 ± 2.30	0.824	7.86 ± 1.39	8.00 ± 1.73	0.863	12.50 ± 2.12	10.10 ± 3.56	0.345	8.00 ± 1.41	7.36 ± 2.41	0.711	8.00 ± 1.41	8.19 ± 3.73	0.942
Haematoma	11.20 ± 1.30	10.26 ± 3.79	0.578	8.80 ± 3.11	7.69 ± 2.30	0.287	7.00 ± 1.00	7.87 ± 1.39	0.164	10.33 ± 4.41	10.11 ± 3.56	0.882	8.50 ± 2.25	7.35 ± 2.41	0.249	8.33 ± 1.63	8.19 ± 3.74	0.926
Wound secretion	9.00 ± 1.82	10.28 ± 3.78	0.501	6.00 ± 2.94	7.72 ± 2.30	0.139	8.50 ± 1.73	7.86 ± 1.38	0.358	9.16 ± 5.38	10.13 ± 3.54	0.511	7.00 ± 2.52	7.37 ± 2.41	0.707	7.33 ± 1.21	8.20 ± 3.74	0.571

*TKA* total knee arthroplasty, *THA* total hip arthroplasty *PSQI*, the Pittsburgh Sleep Quality Index, *ESS* Epworth Sleepiness Scale, *DVT* deep vein thrombosis, *CMVT* calf muscular vein thrombosis

## Discussion

The present study is the first to report the correlation between preoperative sleep parameters and postoperative function. This prospective single-center study revealed that (1) preoperative sleep quality was significantly related to postoperative pain, function, LOS, and analgesic medication consumption following total hip and knee arthroplasty and (2) preoperative sleep parameters did not affect the postoperative complication rate or patient satisfaction scores. Therefore, we believe that preoperative sleep quality can significantly affect postoperative outcomes. Improvements in sleep quality before joint replacement can effectively relieve postoperative pain, improve patient function, and reduce the LOS and analgesic consumption.

Studies have studied the impact of different inventions on postoperative sleep quality [9–11]. Gong et al. examined the effects of sleep quality on early recovery after total knee arthroplasty and noted that patients taking zolpidem achieved significant improvement in sleep and higher levels of satisfaction than those who did not. Patients in the zolpidem group had lower pain scores and took fewer antiemetics than those in the control group. Moreover, a significant correlation between sleep quality and range of motion (ROM) was detected [10]. As such, lowering the incidence of sleep disturbance has the potential to decrease pain and enhance a patient’s mental status during daytime hours after TJA, which may improve functional outcomes and hasten postoperative recovery [28]. However, Gong et al. studied the relationship between postoperative sleep quality and postoperative rehabilitation and did not further explore the influence of preoperative sleep quality in patients. Kirksey et al. explored sleep disruption after total knee arthroplasty and the impact of melatonin on sleep and postoperative pain, and exogenous melatonin did not appear to improve postoperative sleep or pain to a significant degree [11].

The dynamic relationship between chronic sleep deficiency and pain thresholds is complicated. However, it is well established that chronic pain conditions are often associated with insufficient sleep, which in turn is a predictor of persistent pain symptoms in both general populations and in individuals with chronic pain [29]. Haack et al. [30] conducted a study and found that activation of the prostaglandin E2 (PGE2) system appeared to be a potential mediator of increased spontaneous pain in response to insufficient sleep. Weingarten et al. conducted a population-based study [31], and the authors concluded that short TSTs were associated with reports of moderate to severe pain. Odegard et al. [32] concluded that sleep restriction reduced the central nervous system (CNS) response to pain, while some of the subjective pain measures indicated hyperalgesia. Recently, Roehrs et al. conducted [15], on the 3- to 4-day preoperative inpatient recovery measures, the sleep extension group reported significantly less average daily pain (4.4 vs. 5.6, *p* < 0.04) and less daily morphine milligram equivalent intake (20.3 vs. 38.6 mg, *p* < 0.02) than the control group [15]. In addition, the PSQI scores were significantly correlated with nocturnal and active pain scores and the consumption of analgesics in the early period after surgery. Our results suggested that a decrease in preoperative sleep quality can affect postoperative pain, increase postoperative pain sensitivity, and increase postoperative analgesic consumption.

The limited ROM after joint replacement is largely due to contributions of postoperative pain, and the postoperative pain threshold is affected by sleep quality. Gong et al. found that the ROM in the zolpidem group was significantly better than that in the control group from POD5 to POD14 [10]. Long et al. compared the ROM after TKA among patients with four forms of insomnia and noted significant differences in active ROM (*p* < 0.001) among patients with the various forms of insomnia (difficulty in staying asleep and non-restorative sleep has increased active ROM in comparison with patients with difficulty in falling asleep and too early awakening) [33]. In the current study, we found that the ROM on POD1 was significantly positively correlated with sleep parameters, but the ROM on POD3 was not related to sleep parameters. A possible cause for the lack of a correlation with ROM on POD3 is that all patients in our medical center were required to try to achieve 110° ROM and were discharged home on POD3.

The LOS continues to be a major predictor of cost for TJA [34]. The present study demonstrated that better preoperative sleep quality can significantly reduce the LOS for TJA. In addition, Halawi et al. demonstrated that a high preoperative pain level was also a significant multivariable predictor of hospitalization extending beyond 2 days (*p* = 0.001) [35]. Long et al. compared the LOS after TKA among individuals with one of four forms of insomnia (i.e., difficulty in falling asleep, difficulty in staying asleep, non-restorative sleep, too early awakening) and noted that high scores on “Too early awakening” can cause a significantly longer LOS among individuals with various forms of insomnia [33]. The results of our current study indicated that poor preoperative sleep quantity can decrease the postoperative pain threshold, and therefore, the patients required more time to recover from surgical trauma and that these patients had a longer LOS.

There are several limitations to be noted in this study: (1) sleep parameters in the present study were all evaluated via subjective instruments. However, compared to studies using objective measures such as polysomnography (PSG), subjective assessment methods can assess patients’ sleep parameters that are closer to their true feelings [33]. (2) Although this is a prospective cohort study, we did not calculate the sample size a priori. We included approximately 500 joint replacements in each group, which is the largest study to research the effects of sleep on total joint replacement.

## Conclusion

Our data revealed correlations between preoperative sleep parameters and postoperative clinical outcomes (i.e., pain, ROM, function, and LOS) in a prospective TJA cohort of 994 patients. Our results indicated that a better sleep quality before the operation could improve the pain threshold, decrease the pain score and analgesic consumption, and reduce the LOS. Considering these proven benefits, we suggest that clinicians should assess the sleep complaints of patients and improve their sleep quality before operation. In addition, these findings point to the importance of and the need for further and larger perspective randomized studies that apply various interventions to improve patients’ sleep health during patient preparation in the preoperative period.

## Data Availability

The datasets analyzed during the current study are available from the corresponding author on reasonable request.

## Abbreviations

BMI: Body mass index
CMVT: Calf muscular vein thrombosis
COPD: Chronic obstruct pulmonary disease
DVT: Deep vein thrombosis
ESS: Epworth Sleepiness Scale
HHS: Harris hip score
KSS: Knee Society Score
LOS: Length of stay
PE: Pulmonary embolism
POD: Postoperative day
POM: Postoperative month
PSQI: Pittsburgh Sleep Quality Index
ROM: Range of motion
THA: Total hip arthroplasty
TKA: Total knee arthroplasty
VAS: Visual analog scale
